# The global clinical trial landscape of PD-1-containing bispecific antibodies for gastric cancer: current status and future directions of targeting immune tolerance

**DOI:** 10.3389/fimmu.2026.1866067

**Published:** 2026-06-22

**Authors:** Ziyue Sha, Zhengzheng Ji, Lanqing Zhao, Song Wang, Zhanjun Guo

**Affiliations:** 1 Department of Immunology and Rheumatology, The Fourth Hospital of Hebei Medical University, Shijiazhuang, China; 2 Department of Geriatrics, The Fourth Hospital of Hebei Medical University, Shijiazhuang, China

**Keywords:** biomarker, bispecific antibody, clinical trial landscape analysis, gastric cancer, PD-1

## Abstract

Gastric cancer (GC) remains a leading cause of cancer-related death worldwide, and PD-1 monotherapy offers limited clinical benefit due to intrinsic and acquired immune tolerance. PD-1-containing bispecific antibodies (BsAbs) represent an emerging immunotherapeutic strategy to reverse immune suppression, enhance antitumor immunity, and overcome therapy resistance. Here, we present a systematic clinical trial landscape analysis to characterize the global development of PD-1-based BsAbs for GC. We identified 121 interventional clinical trials registered up to April 17, 2026, from the INFORMA database. Total trial numbers grew continuously from 2017 to 2025, with phase II trials constituting the largest segment. The most frequent non-PD-1 cotarget was CTLA-4, followed by VEGF, TIGIT, and LAG3. Most trials combined BsAbs with chemotherapy, focused on stage III/IV GC, and were conducted in the first-line or neoadjuvant setting. Academic institutions sponsored the majority of trials, and 84.30% were performed in China. Further temporal regression revealed annual growth for CTLA-4, VEGF and TIGIT-targeted programs, and correlation analyses indicated divergent co-target preferences across distinct sponsors and trial status and combination therapy. Subgroup comparison identified that combination therapy was more common in China-led trials, whereas monotherapy was preferentially adopted in non-China-led trials. This Perspective delineates a rapidly expanding global pipeline with China as the leading contributor. Our findings support the rational design of PD-1 BsAbs and combination strategies to target immune tolerance in GC, and highlight future priorities including balancing therapeutic efficacy against safety risks, biomarker-driven patient stratification, and international collaboration to optimize immunotherapy.

## 1 Introduction

Gastric cancer (GC) is among the most prevalent and lethal malignancies worldwide, representing a substantial public health burden with unmet therapeutic needs, especially for advanced disease (1). Immune checkpoint inhibitors (ICIs) targeting the PD-1/PD-L1 axis have reshaped the treatment paradigm for GC (2–4). However, low response rates and frequent therapeutic resistance remain major obstacles, largely driven by tumor-induced immune tolerance and immunosuppressive tumor microenvironments.

PD-1-containing bispecific antibodies have emerged as a promising next-generation approach to simultaneously block PD-1 and complementary immunosuppressive pathways, thereby enhancing antitumor immunity, reversing immune tolerance, and overcoming resistance. Amid rapid global development, a comprehensive landscape analysis is urgently needed to summarize trends, guide rational design, and inform future research.

In this Perspective, we systematically analyze 121 interventional clinical trials to depict the global developmental landscape of PD-1-based bispecific antibodies for GC, with emphasis on targeting immune tolerance and improving clinical outcomes.

## 2 Materials and methods

### 2.1 Data source and selection criteria

A systematic search was performed using the INFORMA database (https://www.citeline.com/en/products-services/clinical/trialtrove) as of April 17, 2026, to identify all interventional clinical trials evaluating PD-1-containing bispecific antibodies in patients with histologically confirmed GC. The predefined search strategy was constructed as: Drug Type: “Bispecific antibody” AND Target: “programmed cell death 1” AND Therapeutic area: “Oncology” and Disease: “Gastric”.

### 2.2 Inclusion and exclusion criteria

Eligibility criteria were strictly defined as follows: Inclusion criteria: a. interventional clinical trial; b. GC as the primary indication, c. investigational agents consisting of bispecific antibodies incorporating PD-1 as one target. Exclusion criteria: a. non-interventional studies (e.g., observational, registry, case reports); b. trials focused on non-gastric malignancies; c. monospecific PD−1/PD−L1 inhibitors or combination of two monoclonal antibodies; d. duplicate registrations or trials with no available data. Duplicate trials were identified according to trial title, target agent, combination therapy, patient characteristics and sponsor; only the most complete record was retained. A flow diagram summarized the study selection process was shown as **Supplementary Figure 1**.

### 2.3 Data extraction

Two independent reviewers conducted dual data extraction and manual adjudication of unstructured trial metadata, with discrepancies resolved by consensus and all decisions documented in a standardized audit trail to ensure data accuracy and reproducibility. This rigorous methodological framework adheres to the recently published TITAN Guidelines 2025 for high-quality clinical trial landscape analyses (5). Analyzed endpoints included temporal trial trends, development phases, trial status, non-PD-1 co-target distribution, combination therapeutic strategies, patient disease stage, treatment line settings, sponsors, and geographic distribution.

### 2.4 Statistical analysis

Categorical variables were presented as frequencies and percentages. Poisson regression was applied to assess annual temporal trends. Between−group comparisons were performed using the Pearson χ² test or Fisher’s exact test where appropriate. *P* ≤ 0.05 was considered statistically significant. All statistical analyses were performed using Python 3.11.

## 3 Results

### 3.1 Trial characteristics

**Figure 1:**
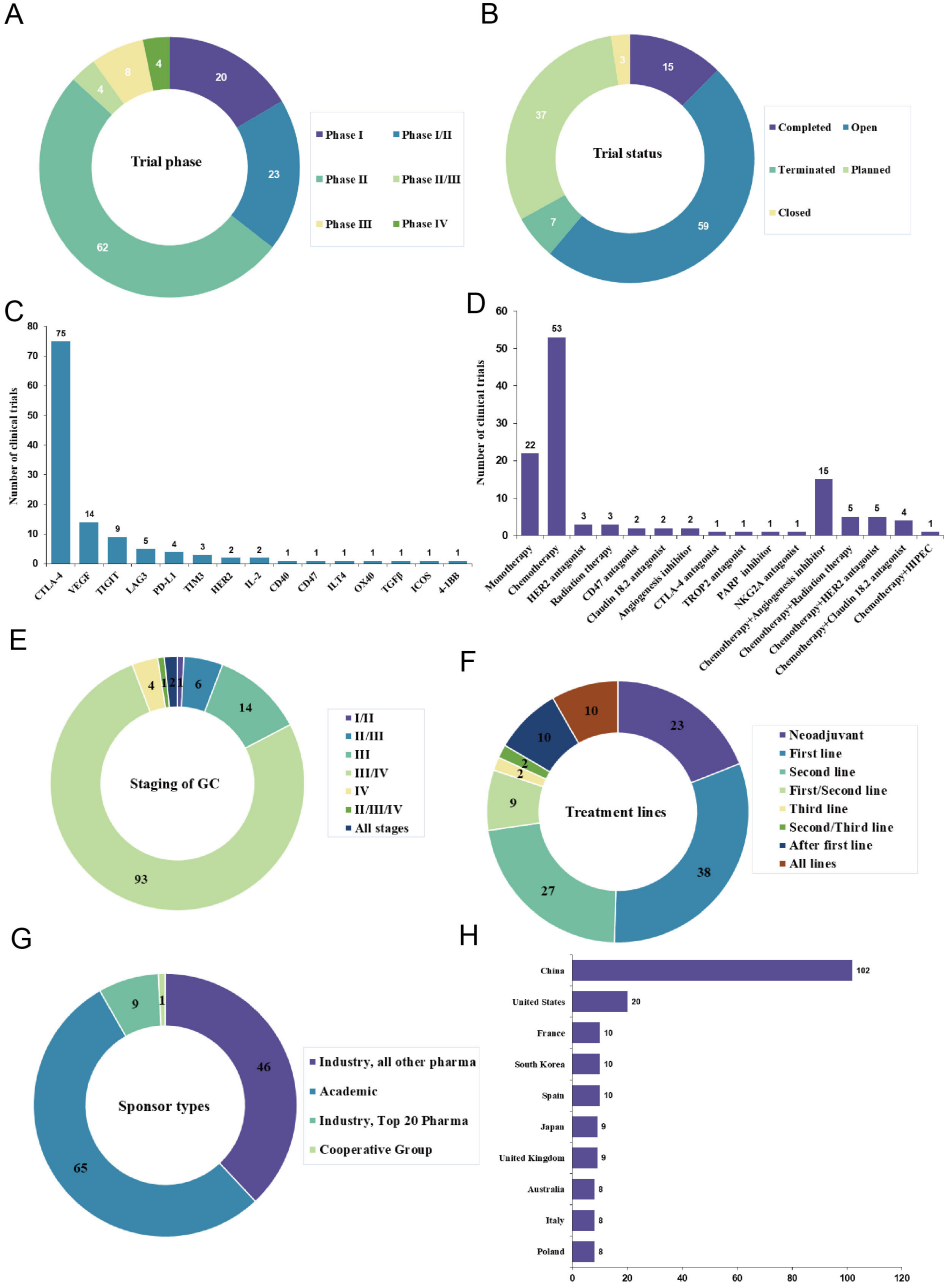
Landscape of clinical trials evaluating PD-1-containing bispecific antibodies in gastric cancer. **(A)** Distribution of trial phase; **(B)** Distribution of trial status; **(C)** Non-PD-1 co-targets of bispecific antibodies under clinical investigation; **(D)** Single and combined usage of bispecific antibodies; **(E)** Patient disease stage distribution; **(F)** Treatment line distribution; **(G)** Sponsor types distribution. **(H)** Geographic distribution of trials (Top 10).

Our initial search yielded 121 clinical trials meeting the inclusion criteria (**Supplementary Table 1**). Phase II trials constituted the largest proportion of the developmental pipeline (51.24%), primarily focused on evaluating safety profiles and preliminary anti-tumor efficacy (**Figure 1A**). Trial status assessment demonstrated 59 open, 37 planned, 15 completed, 7 terminated, and 3 closed studies (**Figure 1B**), indicating a highly active clinical landscape with a favorable developmental risk profile. Regarding target selection, CTLA-4 was the most common non-PD-1 co-target (61.98%), followed by VEGF (11.57%), TIGIT (7.44%), LAG3 (4.13%), PD-L1 (3.31%), and TIM3 (2.48%) (**Figure 1C**). Combination therapy dominated clinical deployment, with PD-1-based bispecific antibodies most frequently investigated in combination with chemotherapy (**Figure 1D**), supporting a strategic paradigm of immunochemotherapy synergy to improve clinical outcomes. Patient enrollment was highly concentrated in stage III/IV disease (76.86%), with stage III and stage II/III accounting for 11.57% and 4.96%, respectively (**Figure 1E**). With respect to treatment lines, first-line evaluation was most prevalent (31.40%), followed by second-line (22.31%) and neoadjuvant applications (19.01%) (**Figure 1F**). These patterns illustrate a clinically oriented development pathway that prioritizes advanced systemic therapy while gradually moving toward earlier disease stages. In terms of sponsor types, academic institutions funded 53.72% of the trials (**Figure 1G**). Geographically, 84.30% of trials were conducted in mainland China, followed by the United States (16.53%), France (8.26%), South Korea (8.26%), and Spain (8.26%) (**Figure 1H**). This pronounced geographic concentration aligns with the high global incidence and mortality of GC in East Asia, reflecting regional disease burden and unmet clinical demand.

### 3.2 Temporal trends analysis

**Figure 2:**
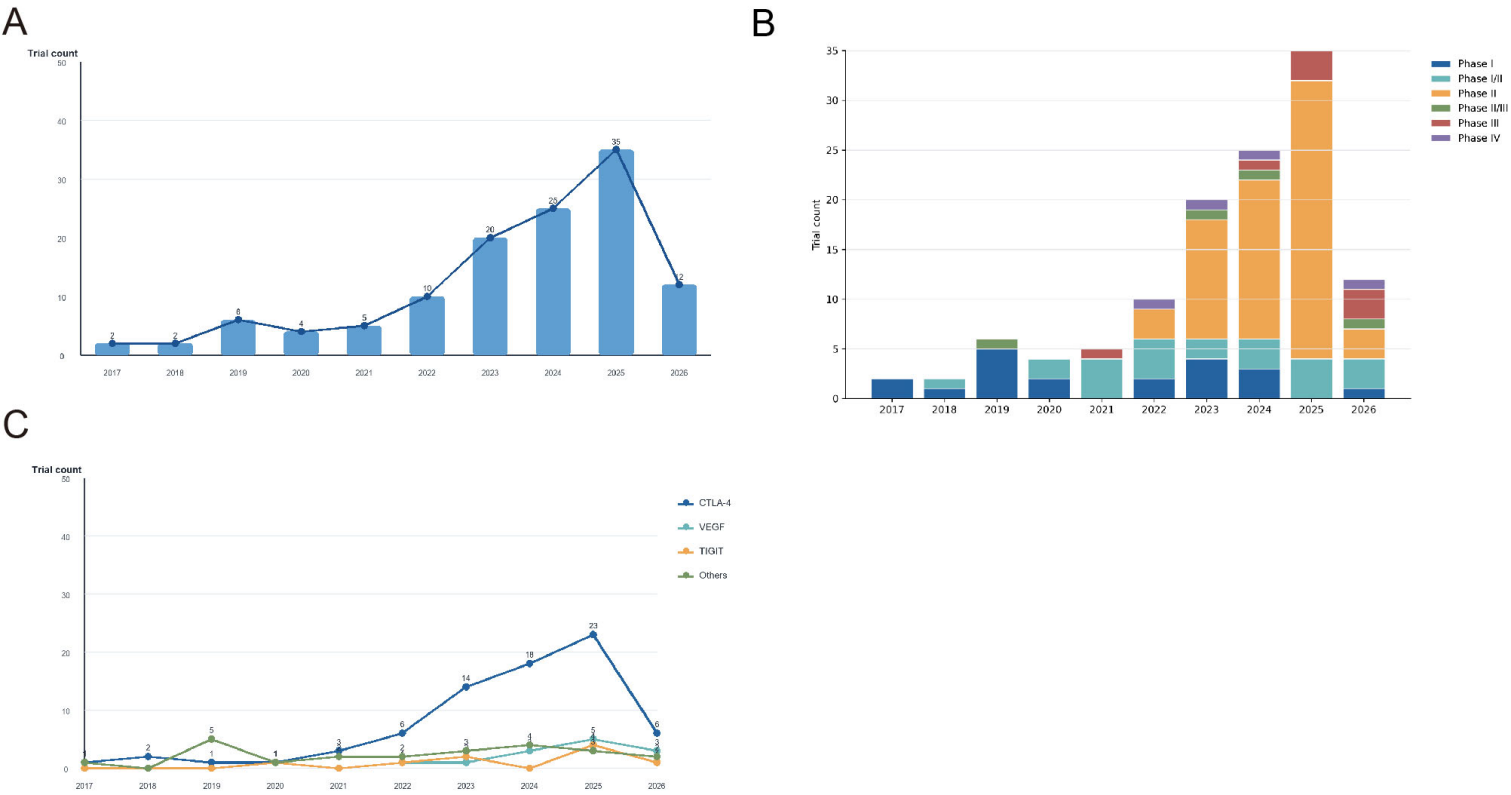
Temporal trends analysis. **(A)** Annual trend of total trial counts; **(B)** Trial counts by phase over time; **(C)** Trends of major co-targets over time.

To characterize the dynamic evolution of PD-1-containing bispecific antibody clinical development, we performed temporal trend analysis on trial registration data from 2017 to 2025. Overall trial numbers displayed a statistically significant rising tendency from 2 in 2017 to 35 in 2025 (**Figure 2A**) (coefficient = 0.3837, *P* < 0.001, **Supplementary Table 2**). Phase-specific analyses revealed disparate annual trends (**Figure 2B**): phase II trials presented the most prominent ascending trend (coefficient = 0.7853, *P* < 0.001, **Supplementary Table 2**), followed by phase III (coefficient = 0.6837, *P* = 0.0358, **Supplementary Table 2**) and phase I/II trials (coefficient = 0.2240, *P* = 0.0189, **Supplementary Table 2**). Such divergent trends might imply a gradual field shift away from early-phase safety-only testing toward intermediate-stage efficacy exploration. In terms of co-target development (**Figure 2C**), CTLA-4-targeted trials grew significantly year by year (coefficient = 0.4560, *P* < 0.001, **Supplementary Table 2**); meanwhile, emerging targets including VEGF (coefficient = 0.5973, *P* = 0.0024, **Supplementary Table 2**) and TIGIT (coefficient = 0.4806, *P* = 0.0159, **Supplementary Table 2**) also achieved statistically significant annual increments. These shifting patterns suggest a potential gradual expansion from single dual-checkpoint designs toward diversified combinatorial strategies.

### 3.3 Co-target correlation analysis

We further performed cross-tabulation correlation analyses to explore the associations between non-PD-1 co-target preferences and other trial characteristics. No statistically significant correlation was detected between co-target category and trial phase allocation (*χ²* = 74.4604, *P* = 0.3353, **Supplementary Figure 2A**), despite visible numerical clustering: CTLA-4-centered programs predominated within Phase II trials (*n* = 48), whereas most novel emerging targets (including OX40, CD47, ILT4 and TGF-β) were only explored in early Phase I trials. By contrast, co-target distribution significantly correlated with trial status (*χ²* = 80.5686, *P* = 0.0174, **Supplementary Figure 2B**): CTLA-4-related trials occupied the largest proportion of both open (*n* = 34) and planned (*n* = 29) cohorts, while relatively few CTLA-4 programs were terminated or closed; most rare novel-target studies either remained in early open enrollment or were terminated. According to the Sankey diagram mapping co-target to treatment modalities, co-target distribution showed a statistically significant correlation with therapeutic strategy selection (*χ²* = 271.8074, *P* = 0.0159, **Supplementary Figure 2C**). CTLA-4 constituted the dominant co-target across most regimen subtypes, whereas multiple less-frequent co-targets were more often tested in monotherapy settings. Sponsor-type stratification revealed a strong statistically significant correlation with co-target selection (*χ²* = 97.3785, *P* < 0.001, **Supplementary Figure 2D**). Academic sponsors overwhelmingly prioritized CTLA-4 development (*n* = 55); top 20 pharmaceutical companies tended to focus on TIGIT, while non-top 20 industrial sponsors diversified their pipeline across LAG3, PD-L1, TIM3 and multiple novel immune targets.

### 3.4 Subgroup comparison between China-led and non-China-led trials

Given the substantial predominance of China-led trials, which accounted for 102 of the 121 registered trials (84.30%), a prespecified subgroup analysis was conducted to compare baseline characteristics between China-led and non-China-led trials (**Supplementary Table 3**). No statistically significant differences were observed in trial phase distribution (*P* = 0.462), co-target selection including CTLA-4 (*P* = 0.153) and VEGF partners (*P* = 0.232), enrolled patient disease stage (*P* = 1.000), allocation of neoadjuvant/first-line treatment regimens (*P* = 0.198), or sponsor composition (*P* = 0.108). Although the completion rate was numerically higher among non-China-led trials (26.32% vs. 9.80% in China-led trials), this difference did not reach statistical significance (*P* = 0.060). The sole statistically significant divergence pertained to therapeutic modality (*χ²* = 8.672, *P* = 0.007): combination therapy was markedly predominant in China-led trials (86.3%), whereas monotherapy was substantially more common in non-China-led trials (42.1% vs. 13.7% in China-led cohorts).

## 4 Discussion

Completed trials included in this analysis have delivered encouraging clinical signals that validate the developmental rationale of PD-1-containing bispecific antibodies in GC. Notably, the phase III COMPASSION-15 trial demonstrated that cadonilimab, a PD-1/CTLA-4 bispecific antibody, combined with chemotherapy significantly improved progression-free survival and overall survival compared with chemotherapy alone in the first-line treatment of advanced GC or gastroesophageal junction adenocarcinoma (6). These findings only provide preliminary evidence favoring dual checkpoint combination rather than universal clinical superiority of dual-targeted strategy. The predominance of phase II trials reflects a risk-mitigating developmental strategy, emphasizing the optimization of dose, combination partners, and patient populations prior to large-scale phase III registration studies. Since bispecific antibodies broadly engage and activate T cells, they carry an inherent risk of on-target/off-tumor toxicity, including immune-related adverse events. Although terminated/closed trials are few (8.26%), systematic meta-analysis of safety and efficacy across all trials is currently lacking, limiting definitive conclusions about universal safety and efficacy profiles. Optimizing the risk-benefit balance while lowering unwanted toxicities remains a key consideration for future trial design.

Preclinical advances have provided mechanistic support for the observed target preferences and clinical strategies. Concurrent blockade of CTLA-4 enhances T-cell priming, reduces regulatory T-cell-mediated immunosuppression, and synergizes with PD-1 inhibition to reinvigorate anti-tumor immune responses (7). Inhibition of VEGF normalizes tumor vasculature, alleviates hypoxia, and promotes immune cell infiltration into the tumor microenvironment, providing a strong biological rationale for PD-1/VEGF bispecific approaches (8). Emerging preclinical data also highlight TIGIT and LAG3 as key mediators of primary and acquired resistance to PD-1 monotherapy, justifying their ongoing clinical evaluation as co-targets in bispecific formats (9). Collectively, these mechanistic insights align with and validate the target distribution observed in clinical trials.

Notably, PD-1-based bispecific antibodies offer distinct advantages over combination therapy with two monoclonal antibodies. As a single agent, bispecific antibodies avoid pharmacokinetic mismatch and heterogeneous tissue distribution (10, 11). Synchronized targeting enhances local immune activation while reducing systemic immune-related adverse events, balancing efficacy and safety (12). Bispecific antibodies also streamline clinical development, simplify dosing, lower production costs, and improve patient adherence, supporting their translational potential (11, 13).

Biomarker-guided enrollment has become indispensable for optimizing precision immunotherapy and refining trial design of future bispecific antibody trials in GC. PD-L1 expression and mismatch repair deficiency (dMMR) are clinically validated predictive biomarkers for ICI response, with dMMR conferring high sensitivity to PD-1 blockade (14, 15). Emerging candidates include tumor mutational burden (TMB) and Epstein–Barr virus (EBV) positivity (16, 17). Notably, combining trastuzumab with PD-1 inhibitors in HER2-positive GC enhances antitumor immunity beyond HER2-targeted effects, which suggests HER2 status may serve not only as a therapeutic target but also as a contextual modulator of ICI response (18). Similarly, recent data show that 13–22% of CLDN18.2-positive GC tumors co-express PD-L1 (CPS ≥5) (19, 20), and anti-CLDN18.2 antibodies induce immune-mediated cytotoxicity, indicating CLDN18.2 expression may enrich ICI-responsive subsets (21). Moreover, comprehensive profiling of intratumoral immune features (such as CD8^+^ T-cell density, tertiary lymphoid structure presence) enables classification into immune-hot and immune-cold phenotypes to guide personalized bispecific selection (17, 22, 23). Although biomarker-enriched trial design theoretically improves cohort screening and trial efficiency, most ongoing trials still adopt unselected all-comer enrollment, leaving considerable room for future optimization.

China-led trials account for 84.30% of all included studies, and such geographic concentration is driven by multiple interrelated factors. First, China bears nearly 40% of the global GC incidence and mortality, creating an acute clinical need for novel therapies (1). Second, China’s National Medical Products Administration (NMPA) has established accelerated approval pathways including breakthrough designation, conditional approval and priority review pathways, which markedly shorten the clinical-to-regulatory timeline for innovative bispecific drugs, distinguishing its framework from Western regulators (24). Finally, benefiting from national strategies prioritizing innovative immunotherapy, China’s thriving domestic biotech industry and complete clinical trial infrastructure together with lower trial running costs greatly promote the rapid launch of relevant clinical projects. In contrast, lower GC incidence, higher trial expenditures, and differing commercial priorities constrain trial activity in Western regions. Subgroup analyses reveal comparable core trial design between China-led and non–China-led trials, with therapeutic regimen serving as the sole significant discrepancy: combination therapy dominates in China-led trials, whereas monotherapy prevails in non–China-led studies. This difference reflects divergent clinical development philosophies, and ethnic variations in GC genetics and immune microenvironment (25), which may limit extrapolating existing data to Western patients, and underscore the need for international cooperative trials.

Several inherent limitations exist for this landscape analysis. First, most enrolled trials are early-phase, with immature survival endpoints and inadequate follow-up to confirm durable treatment benefits. Second, bispecific antibodies may raise potential risks of excess immune-related adverse events and require sustained safety monitoring. Third, no validated biomarkers can reliably stratify GC patients to predict bispecific antibody responsiveness across different subgroups. Finally, preferential publication of positive outcomes may introduce publication bias and overestimate the real-world efficacy of these investigational drugs.

In conclusion, this comprehensive landscape analysis reveals a dynamic, rapidly expanding clinical development ecosystem for PD-1-containing bispecific antibodies in GC based on clinical trial data with supplementary temporal and correlation statistics. We found that trial numbers have steadily increased. Co-target correlation data reveals divergent co-target preferences across sponsors, trial status and combination therapy. China dominates global clinical development, and cross-regional subgroup comparison reveals divergent preference between combination and monotherapy despite analogous core trial design. From a forward-looking perspective, three pivotal directions deserve priority in subsequent translational exploration: first, balancing therapeutic efficacy against off-target toxicity remain key priorities for future trial design; second, implementing biomarker-stratified trial designs to improve patient selection and trial efficiency; third, promotion of international collaborative trials to reduce geographic disparities. Continued clinical and translational investigation is warranted to fully realize the potential of PD-1-based bispecific antibodies and improve survival outcomes for patients with GC worldwide.

## Data Availability

The raw data supporting the conclusions of this article will be made available by the authors, without undue reservation.
