# Supplementary material for: The global clinical trial landscape of PD-1-containing bispecific antibodies for gastric cancer: current status and future directions of targeting immune tolerance
Source: Front Immunol. 2026 Jun 22;17:1866067. doi: 10.3389/fimmu.2026.1866067 (PMC13333607; doi:10.3389/fimmu.2026.1866067)
Supplement: Supplementary file 1 [file Table1.docx]

**Supplementary Table S1** The characteristics of 121 clinical trials

|  | **Trial ID** | **Trial Title** | **Bispecific Antibody** | **Co-target** | **Combination therapy** | **Start Date** | **Trial Phase** | **Trial Status** | **Patient stage** | **Treatment line** | **Sponsor Type** | **Trial Countries** |
| --- | --- | --- | --- | --- | --- | --- | --- | --- | --- | --- | --- | --- |
| 1 | 2017-0696 CP-MGD013-01 CTR20210077 EudraCT Number: 2018-003153-21 J17122 NCI-2018-00122 NCT03219268 TrialTroveID-305380 | A Phase I, First-in-Human, Open-Label, Dose Escalation Study of MGD013, A Bispecific DART Protein Binding PD-1 and LAG-3 in Patients With Unresectable or Metastatic Neoplasms | MGD013 | LAG3 | HER2 antagonist | 2017-8-18 | I | Completed | III/IV | Second line/Third line/Fourth Line or greater | Industry, all other pharma | Australia; Bulgaria; China; Poland; Spain; Thailand; Ukraine; United States |
| 2 | AK 104-101 AK104-101 COMPASSION-01 NCT03261011 TrialTroveID-307717 | A Phase IA/IB Multicenter, Open-label, Dose-escalation, and Dose-expansion Study to Evaluate the Safety, Pharmacokinetics, and Antitumor Activity of AK104 in Subjects With Advanced Solid Tumors. | Cadonilimab | CTLA-4 | monotherapy | 2017-10-3 | I | Completed | III/IV | First line | Industry, all other pharma | Australia |
| 3 | 2018-0257 20182093 AAAR8229 DUET-2 NCI-2018-00935 NCT03517488 S18-00049 TrialTroveID-283886 XmAb20717-01 | A Phase I Multiple Dose Study to Evaluate the Safety and Tolerability of XmAb20717 in Subjects With Selected Advanced Solid Tumors | XmAb20717 | CTLA-4 | monotherapy | 2018-7-10 | I | Completed | III/IV | Second line/Third Line/Fourth Line or greater | Industry, all other pharma | United States |
| 4 | AK104-201 COMPASSION-03 COMPASSION-04/AK104-201 CTR20182027 NCT03852251 TrialTroveID-340045 | A Multicenter, Open-label, Phase Ib/II Study of AK104, a PD-1/CTLA-4 Bispecific Antibody, in Subjects with Advanced Solid Tumors or AK104 in Combination with Oxaliplatin and Capecitabine As First-line Therapy in Subjects with Advanced Unresectable or Metastatic Gastric or Gastroesophageal Junction Adenocarcinoma. | Cadonilimab | CTLA-4 | Chemotherapy | 2018-12-21 | I/II | Completed | I/II/III/IV | First line | Industry, all other pharma | China |
| 5 | CIBI318A101 CTR20190340 NCT03875157 TrialTroveID-342666 | To Evaluate the Safety, Tolerability, and Initial Efficacy of IBI318 in Patients With Advanced Malignancy, Multicenter, IA/IB Study. | IBI318 | PD-L1 | monotherapy | 2019-4-19 | I | Completed | III/IV | Second line/Third line/Fourth Line or greater | Industry, all other pharma | China |
| 6 | 122642 (XmAb23104-01 (DUET-3)) 2019-0037 DUET-3 NCI-2019-00828 NCT03752398 PRO00102372 REFMAL 649 DDU - XmAb23104-01 RG1004763 TrialTroveID-274817 XmAb23104-01 | A Phase I Multiple-Dose Study to Evaluate the Safety and Tolerability of XmAb23104 in Subjects With Selected Advanced Solid Tumor | XmAb23104 | ICOS | CTLA-4 antagonist | 2019-5-1 | I | Completed | III/IV | Second line | Industry, all other pharma | United States |
| 7 | 17101 2019-0156 EudraCT Number: 2018-003871-37 J1E-MC-JZEA NCI-2019-03043 NCT03936959 TrialTroveID-348817 | A Phase 1 Study of LY3434172, a Bispecific Antibody Monotherapy in Advanced Solid Tumors | IBI318 | PD-L1 | monotherapy | 2019-5-24 | I | Terminated | III/IV | Second line | Industry, Top 20 Pharma | Australia; Belgium; France; South Korea; United States |
| 8 | COMPASSION-37 COMPASSION-37/AK104-311 TrialTroveID-694288 | A Global Multicenter Phase III Trial Evaluating Cadonilimab (PD‑1/CTLA‑4 Bispecific Antibody) Plus Chemotherapy Versus Chemotherapy With or Without Nivolumab as First‑Line Treatment for HER2‑Negative, Unresectable or Metastatic Gastric or Gastroesophageal Junction Adenocarcinoma. | Cadonilimab | CTLA-4 | Chemotherapy | 2026-3-30 | III | Planned | IV | First line | Industry, all other pharma | China |
| 9 | CTR20192299 HX009-I-01 NCT04097769 TrialTroveID-357721 | A Phase I, First-in-Human Study Evaluating the Safety, Tolerability, and Initial Efficacy of HX009 in Patients With Advanced Malignancies | HX009 | CD47 | monotherapy | 2019-6-12 | I | Completed | III/IV | Second line/Third Line/Fourth Line or greater | Industry, all other pharma | Australia; China |
| 10 | 19-1069 MC# 19-17 NCI-2019-05462 NCT03986606 PSB205-001 TrialTroveID-347481 | A Phase I, Open-Label, Dose Escalation and Expansion Study to Evaluate the Safety, Tolerability, Pharmacokinetics and Preliminary Anti-tumor Activity of PSB205 in Patients With Relapsed/Refractory Solid Tumors | PSB205 | CTLA-4 | monotherapy | 2019-7-5 | I | Completed | III/IV | Third line | Industry, all other pharma | United States |
| 11 | 071908 201908202 3G-20-1 CP-MGAH22-06 CTR20200660 EudraCT Number: 2019-004699-21 IRAS ID: 282019 MAHOGANY NCI-2019-07167 NCT04082364 TrialTroveID-342312 | A Phase II/III Trial to Evaluate Margetuximab in Combination With INCMGA00012 and Chemotherapy or MGD013 and Chemotherapy in Patients With Metastatic or Locally Advanced, Treatment-naive, HER2-Positive Gastric or Gastroesophageal Junction Cancer | MGD013 | LAG3 | Chemotherapy+HER2 antagonist | 2019-9-30 | II/III | Completed | III/IV | First line | Industry, all other pharma | China; Germany; Italy; Netherlands; Poland; Singapore; South Korea; Spain; Taiwan, China; United Kingdom; United States |
| 12 | CTR20200289 CTR20200393 NCT04178460 TrialTroveID-362079 ZL-2306-006 | A Multicenter, Open-label, Single-arm, Phase Ib Dose Escalation and Multi-cohort Expansion Clinical Study to Assess the Safety and Antitumor Activity of Niraparib in Combination With MGD013 in Patients With Advanced or Metastatic Solid Tumor Who Failed Prior Treatment | MGD013 | LAG3 | PARP inhibitor | 2020-2-3 | I | Terminated | III/IV | Second line/Third Line/Fourth Line or greater | Industry, all other pharma | China; Hong Kong, S.A.R., China |
| 13 | CTR20213371 D967LC00001 DESTINY-Gastric03 DG-03 EUCT number: 2023-504888-16-00 EudraCT Number: 2019-004483-22 jRCT2031200203 NCI-2020-10070 NCT04379596 TrialTroveID-373955 | A Phase Ib/II Multicenter, Open-label, Dose-escalation and Dose-expansion Study to Evaluate the Safety, Tolerability, Pharmacokinetics, Immunogenicity, and Antitumor Activity of Trastuzumab Deruxtecan (T-DXd) Monotherapy and Combinations in Adult Participants With HER2-expressing Gastric Cancer (DESTINY-Gastric-03) | rilvegostomig | TIGIT | HER2 antagonist | 2020-6-3 | I/II | Open | III/IV | First line/Second line | Industry, Top 20 Pharma | Brazil; Canada; China; Germany; Italy; Japan; Netherlands; Poland; Russia; South Korea; Spain; Taiwan, China; United Kingdom; United States |
| 14 | AK112-102 CTR20201888 NCT04597541 TrialTroveID-386180 | A Phase I/II Trial of AK112 in Advanced Solid Tumor | Ivonescimab | VEGF | monotherapy | 2020-10-21 | I/II | Completed | III/IV | all lines | Industry, all other pharma | China |
| 15 | CTR20201358 NCT04606472 SI-B003-101 TrialTroveID-388055 | Phase I Clinical Study to Evaluate the Safety, Tolerability, Pharmacokinetic Characteristics and Preliminary Efficacy of SI-B003, a PD-1/CTLA-4 Bispecific Antibody, in Patients With Advanced Solid Tumors | SI-B003 | CTLA-4 | monotherapy | 2020-11-10 | I | Closed | III/IV | Second line | Industry, all other pharma | China |
| 16 | CTR20211884 EMB02X101 EPIMAB_EMB02X101 NCT04618393 TrialTroveID-388692 | A Phase I/II Trial of EMB-02, a Bi-specific Antibody Against PD-1 and LAG-3, in Patients With Advanced Solid Tumors | EMB-02 | LAG3 | monotherapy | 2021-3-11 | I/II | Terminated | III/IV | all lines | Industry, all other pharma | Australia; China; United States |
| 17 | AK109-201 CTR20211244 NCT04982276 TrialTroveID-396120 | An Open Label, Multicentre, Phase Ib/II Clinical Study of AK109 and AK104 With or Without Chemotherapy in Second-line Treatment of Advanced Gastric Adenocarcinoma or Gastroesophageal Junction Adenocarcinoma | Cadonilimab | CTLA-4 | Angiogenesis inhibitor | 2021-8-23 | I/II | Open | III/IV | Second line | Industry, all other pharma | China |
| 18 | AK104-302 COMPASSION-15 COMPASSION-15/AK104-302 CTR20211567 NCT05008783 TrialTroveID-408571 | Randomized, Double-Blind, Multi-Center, Phase III Clinical Study A Comparison Of The Effectiveness And Safety Of AK104 Combined With Oxaliplatin And Capecitabine (XELOX) Regimen And Placebo Combined With XELOX Regimen As The First-Line Treatment For Locally Advanced Or Metastatic Gastric Adenocarcinoma Or Gastroesophageal Junction Adenocarcinoma That Is Not Resectable | Cadonilimab | CTLA-4 | Chemotherapy | 2021-9-17 | III | Completed | III/IV | First line | Industry, all other pharma | China |
| 19 | 152970 CTR20230222 D9570C00001 EUCT number: 2022-502774-17-00 EudraCT Number: 2021-000036-57 jRCT2031220675 NCT04931654 TrialTroveID-406761 | A Phase I/IIa Open-label Dose Escalation and Expansion Study to Evaluate the Safety, Pharmacokinetics, Pharmacodynamics, and Efficacy of AZD7789, an Anti-PD-1 and Anti-TIM-3 Bispecific Antibody, in Participants With Advanced or Metastatic Solid Tumors | AZD7789 | TIM3 | monotherapy | 2021-9-28 | I/II | Terminated | III/IV | all lines | Industry, Top 20 Pharma | Canada; China; France; Georgia; Japan; Moldova Republic; Netherlands; New Zealand; Spain; Turkey; United States |
| 20 | AK109-102 CTR20212388 NCT05142423 TrialTroveID-415401 | An Open Label, Multi-center, Phase Ib/II Clinical Study of AK109 Combined With AK104 to Evaluate the Safety, Tolerability, Pharmacokinetics and Anti-tumor Activity in Advanced Solid Tumors | Cadonilimab | CTLA-4 | Angiogenesis inhibitor | 2021-11-3 | I/II | Open | III/IV | Second line | Industry, all other pharma | China |
| 21 | ChiCTR2200057704 TrialTroveID-427603 | An interventional single-center clinical study of the safety, tolerability and efficacy of AK104 intraperitoneal infusion combined with low-dose radiotherapy as second-line or later therapy for locally advanced or metastatic gastric/gastroesophageal junction adenocarcinoma | Cadonilimab | CTLA-4 | Radiation therapy | 2022-1-1 | I/II | Open | III/IV | Second line/Third line/Fourth Line or greater | Academic | China |
| 22 | AK117-202 CTR20212989 NCT05214482 TrialTroveID-401363 | A Phase Ib/II Study of AK112(PD-1 / VEGF Bispecific Antibody) in Combination With AK117(Anti-CD47 Antibody)With or Without Chemotherapy in Advanced Malignant Tumors | Ivonescimab | VEGF | CD47 antagonist | 2022-1-22 | I/II | Completed | III/IV | Third line | Industry, all other pharma | China |
| 23 | CTR20220528 KN052-CHN-001 NCT05309512 TrialTroveID-419806 | A Phase Ia/Ib Clinical Study to Evaluate the Safety, Tolerability, Pharmacokinetics/Pharmacodynamics, and Antitumor Activity of KN052 in Chinese Subjects With Advanced Solid Tumors | KN052 | OX40 | monotherapy | 2022-5-27 | I | Terminated | III/IV | Second line | Industry, all other pharma | China |
| 24 | AK117-204 CTR20220284 NCT05235542 TrialTroveID-401362 | A Phase Ib/II Study of AK104 PD-1 / CTLA-4 Bispecific Antibody and AK117 Anti-CD47 Antibody in Combination With or Without Chemotherapy in Advanced Malignant Tumors | Cadonilimab | CTLA-4 | CD47 antagonist | 2022-7-12 | I/II | Open | III/IV | First line | Industry, all other pharma | China |
| 25 | TrialTroveID-499208 | Real-world outcomes of cadonilimab (PD-1/CTLA-4 bispecific antibody) plus chemotherapy as first-line treatment in advanced gastric (G) or gastroesophageal junction (GEJ) cancer with PD-L1 CPS≤5 | Cadonilimab | CTLA-4 | Chemotherapy | 2022-8-1 | IV | Open | III/IV | First line | Academic | South Korea |
| 26 | CTR20220704 Keyplus-001 Keyplus-01 LB1410-CHN-I-01 NCT05357651 TrialTroveID-406485 | A Phase I, Multicenter, Open-Label Study to Evaluate the Safety, Tolerability, Pharmacokinetics, Pharmacodynamics, and Immunogenicity of LB1410, A Recombinant Anti-PD-1 and Anti-TIM-3 Humanized Bispecific Antibody for Injection in Patients With Advanced Solid Tumors or Lymphoma (Keyplus-001) | LB1410 | TIM3 | monotherapy | 2022-8-12 | I | Open | III/IV | Second line/Third Line | Industry, all other pharma | China |
| 27 | NCT05915481 SCARCE SCARCE-1 TrialTroveID-474841 | Clinical Study of Stereotactic Body Radiotherapy Combined With Cadonilimab for the Treatment of Advanced Refractory Malignant Solid Tumors | Cadonilimab | CTLA-4 | Radiation therapy | 2022-8-28 | I/II | Completed | III/IV | Second line/Third Line/Fourth Line or greater | Academic | China |
| 28 | 2022-0351 23-144 BASEC2022-01839 CTR20222821 D926UC00001 EUCT number: 2023-509436-26-00 EudraCT Number: 2022-000776-19 IRAS ID-1005888 jRCT2031220404 NCI-2022-07990 NCT05489211 SNCTP000005356 TrialTroveID-438958 TROPION- PanTumor03 TROPION-PanTumor03 | A Phase II, Multicentre, Open-label, Master Protocol to Evaluate the Efficacy and Safety of Datopotamab Deruxtecan (Dato-DXd) as Monotherapy and in Combination With Anticancer Agents in Patients With Advanced/Metastatic Solid Tumours | rilvegostomig | TIGIT | TROP2 antagonist | 2022-9-6 | II | Open | III/IV | all lines | Industry, Top 20 Pharma | Canada; China; France; Germany; Italy; Japan; Poland; South Korea; Spain; Switzerland; Taiwan, China; Turkey; United Kingdom; United States |
| 29 | ChiCTR2300070248 TrialTroveID-465345 | Clinical Study of Conversion Therapy of Cadunilimab Combined With FLOT in Patients With Inoperable Locally Advanced and Metastatic Gastric Cancer | Cadonilimab | CTLA-4 | Chemotherapy | 2022-11-11 | II | Open | III/IV | First line | Academic | China |
| 30 | ChiCTR2200066893 TrialTroveID-484619 | The Efficacy and Safety of Cadonilimab (AK-104) Combined with FLOT Regimen as an Neoadjuvant Therapy for Locally Advanced Gastric/Gastroesophageal Junction Adenocarcinoma: A Prospective, Multicenter, Open Phase II Single Arm Clinical Study | Cadonilimab | CTLA-4 | Chemotherapy | 2022-12-23 | II | Open | III | Neoadjuvant | Academic | China |
| 31 | GC-MATCH NCT05608785 TrialTroveID-447311 | Single-center, Multi-cohort Exploratory Phase Ib/II Clinical Study of First-line Treatment of Unresectable Locally Advanced/Advanced Adenocarcinoma of the Stomach or Gastroesophageal Junction Based on Different Genotypes | BH-2950 | HER2 | Chemotherapy | 2023-1-1 | I/II | Open | III/IV | First line | Academic | China |
| 32 | FUGES-32 NCT06277024 TrialTroveID-505476 | A Study on the Safety and Efficacy of the Combination of cadonilimab, Lenvatinib, and SOX Regimen in the Treatment of HER2 Negative Advanced Gastric or Gastroesophageal Junction Adenocarcinoma Patients | Cadonilimab | CTLA-4 | Chemotherapy+Angiogenesis inhibitor | 2023-1-1 | II | Open | III/IV | all lines | Academic | China |
| 33 | CTR20230169 CTR20230170 D7986C00001 EUCT number: 2024-510977-27-00 EudraCT Number: 2022-002840-29 GEMINI-Gastric jRCT2031230074 NCT05702229 TrialTroveID-456008 | An Open-Label, Multi-Drug, Multi-Centre, Phase II Study to Evaluate the Efficacy, Safety, Tolerability, Pharmacokinetics, and Immunogenicity of Novel Combinations in Participants With Locally Advanced Unresectable or Metastatic Gastric or Gastroesophageal Junction Adenocarcinoma | rilvegostomig | TIGIT | Chemotherapy | 2023-1-16 | II | Open | III/IV | First line/Second line | Industry, Top 20 Pharma | China; Japan; South Korea; Spain; Taiwan, China; United Kingdom; United States |
| 34 | E20221047 NCT05853172 TrialTroveID-468971 | Surgical Conversion of Candonilimab (AK104) Combined With Paclitaxel, S-1 and Apatinib for Unresectable Advanced Gastric(G)/Gastroesophageal Junction(GEJ) Cancer | Cadonilimab | CTLA-4 | Chemotherapy+Angiogenesis inhibitor | 2023-3-21 | II | Open | III/IV | all lines | Academic | China |
| 35 | AK104-IIT-C-W-0031 NCT05800080 TrialTroveID-464881 | An Exploratory Study of Immunotherapy Combined With Anlotinib Hydrochloride Capsules and Chemotherapy in Perioperative Treatment of Locally Advanced Novel Coronavirus Infected Gastric Cancer | Cadonilimab | CTLA-4 | Chemotherapy+Angiogenesis inhibitor | 2023-4-1 | II | Planned | III/IV | Neoadjuvant | Academic | China |
| 36 | ChiCTR2300069226 CTR20230047 leadingpharm2022010 NCT06773507 TrialTroveID-552486 | An Open-label, Multicenter, Phase I Clinical Study to Evaluate the Safety, Tolerability, Pharmacokinetic (PK) Characteristics and Preliminary Efficacy of BC008-1A Injection in Subjects with Advanced Solid Tumors | BC008-1A | TIGIT | monotherapy | 2023-4-11 | I | Open | III/IV | Second line | Industry, all other pharma Government | China |
| 37 | CDX585-01 NCT05788484 TrialTroveID-445871 | A Phase I Dose-escalation and Expansion Study of the PD-1 x ILT4 Bispecific Antibody CDX-585 in Patients With Advanced Malignancies | CDX-585 | ILT4 | monotherapy | 2023-5-11 | I | Completed | III/IV | Second line | Industry, all other pharma | United States |
| 38 | ChiCTR2300072695 TrialTroveID-474531 | Efficacy and Safety of Cadonilimab( AK104) Plus SOX as a Neoadjuvant Regimen in Patients with Locally Advanced Gastric Cancer: A single-arm Open-label Phase II Trial | Cadonilimab | CTLA-4 | Chemotherapy | 2023-6-21 | II | Open | III | Neoadjuvant | Academic | China |
| 39 | 2023-0288 EUCT number: 2022-502456-31-00 INCA 33890-101 jRCT2031240610 NCT05836324 TrialTroveID-467785 | A Phase I, Open-Label, Multicenter Study of INCA33890 in Participants With Advanced or Metastatic Solid Tumors | INCA33890 | TGFβ | Chemotherapy+Angiogenesis inhibitor | 2023-7-24 | I | Open | III/IV | First line/Second line | Industry, all other pharma | Denmark; France; Italy; Japan; Spain; Switzerland; United Kingdom; United States |
| 40 | AK104-IIT-C-N1-0036 NCT05974059 TrialTroveID-479999 | Single-arm, Open-label, Single-center Phase II Clinical Study of Cadonilimab Combined With CapeOX Regimen in Perioperative Treatment of Resectable Locally Advanced Gastric Cancer | Cadonilimab | CTLA-4 | Chemotherapy | 2023-8-1 | II | Planned | II/III | Neoadjuvant | Academic | China |
| 41 | ChiCTR2300076761 TrialTroveID-488852 | Efficacy and Safety of Cadonilimab and COX-2 Inhibitor Combined with Oxaliplatin and Capecitabine (XELOX) in Perioperative Therapy for Locally Advanced Resectable Gastric Adenocarcinoma: A Prospective, Single-Center, Randomized Clinical Trial | Cadonilimab | CTLA-4 | Chemotherapy | 2023-8-21 | II/III | Open | II/III | Neoadjuvant | Academic | China |
| 42 | NCT05948449 TrialTroveID-477891 WCH-2023-526 | The Efficacy and Safety of Chemotherapy Combined With Cadonilimab (AK104) in Neoadjuvant Treatment of Locally Advanced Gastric Cancer/Gastroesophageal Junction Adenocarcinoma: A Prospective, Single-arm, Phase II Clinical Study | Cadonilimab | CTLA-4 | Chemotherapy | 2023-9-1 | II | Open | III/IV | Neoadjuvant | Academic | China |
| 43 | CTR20232413 NCT06016062 RC148-C001 TrialTroveID-483405 | A Multi-center Phase I/II Trial to Evaluate the Efficacy and Safety of RC148 As a Single Agent and Combination Therapy in Patients with Locally Advanced Unresectable or Metastatic Malignant Solid Tumors | RC148 | VEGF | monotherapy | 2023-9-7 | I/II | Open | III/IV | Second line/Third Line/Fourth Line or greater | Industry, all other pharma | China |
| 44 | AK104-PAN ChiCTR2300077949 TrialTroveID-493410 | Efficacy and safety of AK104 combined chemotherapy for conversion therapy of advanced gastric/gastroesophageal junction adenocarcinoma with No. 16a2/b1 lymph node metastasis, multicenter, open, single-arm study | Cadonilimab | CTLA-4 | Chemotherapy | 2023-11-1 | I | Open | III/IV | First line | Academic | China |
| 45 | AK104-IIT-025 NCT06118645 TrialTroveID-491459 | An Open, Multicenter, Phase II Study of Cadonilimab Combined With Paclitaxel (Albumin-bound) in the Treatment of Advanced Gastric Adenocarcinoma or Esophagogastric Junction Adenocarcinoma With PD-(L)1 Inhibitors Resistance | Cadonilimab | CTLA-4 | Chemotherapy | 2023-11-1 | II | Terminated | IV | Second line | Academic | China |
| 46 | AK104-219 CTR20232285 NCT05960955 TrialTroveID-479164 | Phase II Clinical Study of Cadonilimab Combination With Chemotherapy With or Without the Anti-CD47 Antibody AK117 Neoadjuvant/Adjuvant Therapy for Resectable Gastric or Gastroesophageal Junction Adenocarcinoma | Cadonilimab | CTLA-4 | Chemotherapy+Angiogenesis inhibitor | 2023-11-13 | II | Open | III | Neoadjuvant | Industry, all other pharma | China |
| 47 | BL-B01D1-SI-B003-201-05 CTR20232531 NCT06008054 TrialTroveID-482546 | A Phase II Clinical Study to Evaluate the Efficacy and Safety of SI-B003 Monotherapy, BL-B01D1+SI-B003 Combination Therapy and BL-B01D1+PD-1 Monoclonal Antibody in Patients With Locally Advanced or Metastatic Esophageal Cancer, Gastric Cancer, Colorectal Cancer and Other Gastrointestinal Tumors | SI-B003 | CTLA-4 | monotherapy | 2023-11-16 | II | Open | III/IV | all lines | Industry, all other pharma | China |
| 48 | ChiCTR2300077477 TrialTroveID-491860 | A Prospective Single-arm Exploratory Clinical Study of the Efficacy and Safety of DS-1 Plus Cadonilimab Adjuvant Chemotherapy in Patients With Single-immune Non-dominant Localized Advanced Gastric Cancer After D2 Radical Resection | Cadonilimab | CTLA-4 | Chemotherapy+HER2 antagonist | 2023-11-20 | II | Completed | III | First line | Academic Industry, all other pharma | China |
| 49 | APHP220097 EUCT Number: 2024-511857-23-00 EudraCT Number: 2022-004122-22 MONAMI NCT06152523 TrialTroveID-494129 | Monalizumab and MEDI5752 in Patients With MSI and/or dMMR Metastatic Cancer | MEDI5752 | CTLA-4 | NKG2A antagonist | 2023-12-1 | II | Planned | III/IV | Second line | Academic | France |
| 50 | ChiCTR2300077868 TrialTroveID-493171 | A Multicenter, Open-label, Phase II Clinical Trial of Chidamide Combined with Immunotherapy and Chemotherapy as Second-line Treatment for Advanced Metastatic Gastric Cancer | Cadonilimab | CTLA-4 | Chemotherapy | 2023-12-1 | IV | Planned | III/IV | Second line | Academic | China |
| 51 | CTR20233091 NCT05979155 NWY001-101 TrialTroveID-480372 | A Multicenter, Non-randomized, Open-label, Multiple-Dose Phase I Study of NWY001, in Subjects With Advanced Solid Tumors | NWY001 | CD40 | monotherapy | 2024-1-5 | I | Open | III/IV | Second line | Industry, all other pharma | China |
| 52 | ChiCTR2400088619 TrialTroveID-532388 | A Phase II Clinical Study of HIPEC Combined with Cadonilimab and SOX Chemotherapy for Conversion Therapy in Patients with Advanced Gastric Cancer with Peritoneal Metastasis | Cadonilimab | CTLA-4 | Chemotherapy+HIPEC | 2024-1-24 | II | Open | III/IV | First line | Academic | China |
| 53 | ChiCTR2400079586 TrialTroveID-499001 | A Prospective, Single-arm, Phase II Clinical Study of the Safety and Efficacy of Sox Combined with Cadonilimab (Ak104) as Neoadjuvant Therapy in Patients with Pd-l1 Negative (Cps <1) Locally Advanced Upper Gastric/Gastroesophageal Junction Cancer | Cadonilimab | CTLA-4 | Chemotherapy | 2024-2-1 | II | Open | II/III/IV | Neoadjuvant | Academic | China |
| 54 | CTR20240110 NCT06221748 RC48-C035 TrialTroveID-501378 | A Randomized, Multicenter, Open-Label, Phase II/III Study to Evaluate the Safety and Efficacy of Disitamab Vedotin Combined With Cadonilimab in Subjects With HER2-expressing Locally Advanced or Metastatic Gastric Cancer and Gastroesophageal Junction Adenocarcinoma Who Have Progressed on First-line Therapy | Cadonilimab | CTLA-4 | Chemotherapy+HER2 antagonist | 2024-2-7 | II/III | Open | III/IV | Second line | Industry, all other pharma | China |
| 55 | IIT-002 NCT04556253 TrialTroveID-384964 | A Multicenter, Open-Label, Phase II Study of Anti-PD-1/CTLA-4 Bispecific Antibody AK104 in the Perioperative Treatment of Locally Advanced Microsatellite Instability/Mismatch Repair Protein-Deficient Gastric and Colorectal Cancers | Cadonilimab | CTLA-4 | monotherapy | 2024-2-28 | II | Closed | III | Neoadjuvant | Academic | China |
| 56 | NCT06310473 NFEC-2023-582 TrialTroveID-508046 | Efficacy and Safety of Neoadjuvant Cadonilimab and Chemotherapy for Locally Advanced Esophagogastric Junction and Gastric Cancer : a Prospective, Open-label, Single-Arm Phase II Study | Cadonilimab | CTLA-4 | Chemotherapy | 2024-3-1 | II | Planned | III | Neoadjuvant | Academic | China |
| 57 | NCT06202716 NFEC-2023-531 TrialTroveID-499633 | Cadonilimab Plus CapeOX as First-Line Treatment for Advanced GC/GEJC With High Tumor Microenvironment Score (TMEscore). | Cadonilimab | CTLA-4 | Chemotherapy | 2024-4-1 | II | Planned | III/IV | First line | Academic | China |
| 58 | AK104-IIT-041 NCT06196697 TrialTroveID-499265 | A Phase II, Single Arm, Single Center Study of Cadonilimab(AK104) in Combination With Ivonescimab(AK112) Plus Chemotherapy(SOX/XELOX) as First-line Treatment for Advanced Gastric (G) or Gastroesophageal Junction (GEJ) Cancer | Cadonilimab | CTLA-4 | Chemotherapy+Angiogenesis inhibitor | 2024-4-10 | II | Open | III/IV | First line | Academic | China |
| 59 | NCT06349967 TrialTroveID-511568 WCH-2023-1592 | Nab-paclitaxel Combined With Cadonilimab (AK104) for the Second-line Treatment of Advanced Gastric Cancer: A Prospective, Multicenter, Single-arm, Phase II Study | Cadonilimab | CTLA-4 | Chemotherapy | 2024-4-30 | II | Planned | III/IV | Second line | Academic | China |
| 60 | CFPG2024 NCT06406426 TrialTroveID-516253 | An Open and Exploratory Study of The Second-line Treatment of Advanced Gastric or Gastroesophageal Junction Adenocarcinoma With Cadonilimab and Fruquintinib Combined With Paclitaxel-albumin | Cadonilimab | CTLA-4 | Chemotherapy+Angiogenesis inhibitor | 2024-5-1 | II | Open | III/IV | Second line | Academic | China |
| 61 | AK109-301 CTR20241225 NCT06341335 TrialTroveID-510695 | A Randomized, Double-blind, Phase III Study Of Cadonilimab (AK104) Plus Pulocimab (AK109) And Paclitaxel Versus Paclitaxel In Patients With Advanced Gastric Or Gastroesophageal Junction Adenocarcinoma Who Failed First-line Immunochemotherapy | Cadonilimab | CTLA-4 | Chemotherapy+Angiogenesis inhibitor | 2024-6-7 | III | Open | III/IV | Second line | Industry, all other pharma | China |
| 62 | CTR20241814 NCT06468358 TrialTroveID-522762 TRIGGERCD8 | A Phase Ib/II, Open, Dose-escalation and Expansion Study to Evaluate LB1410 in Combination With LB4330 in Patients With Advanced or Metastatic Solid Tumors | LB1410 | TIM3 | CLDN18.2 antagonist | 2024-6-19 | I/II | Open | III/IV | Second line/Third Line/Fourth Line or greater | Industry, all other pharma Academic | China |
| 63 | CTR20242328 NCT06446388 QLS31905-202 TrialTroveID-520339 | A Phase II Clinical Study to Evaluate the Efficacy and Safety of QLS31905 and/or QL1706 Combination Chemotherapy for the Treatment of CLDN18.2-Positive Advanced Malignant Solid Tumors | Iparomlimab and Tuvonralimab | CTLA-4 | Chemotherapy+CLDN18.2 antagonist | 2024-6-30 | II | Terminated | III/IV | First line | Industry, all other pharma | China |
| 64 | NCT06492317 S2021-K006-001 TrialTroveID-525349 | A Prospective, Single-center, Phase II Clinical Study of First-line Treatment for HER2 (Human Epidermal Growth Factor Receptor 2) Overexpressing Advanced Gastric Cancer With Disitamab Vedotin in Combination With Cadonilimab | Candonilimab | CTLA-4 | HER2 antagonist | 2024-7-6 | II | Planned | III/IV | First line | Academic | China |
| 65 | AK104-IIT-C-M-0119 NCT06650332 TrialTroveID-540538 | An Open-label, Multicohort Phase I/II Study of the Cadonilimab (AK104) Combination Regimen as First-line Treatment for HER2-expressing, Unresectable Locally Advanced or Metastatic Gastric (G) or Gastroesophageal Junction (GEJ) Cancer | Cadonilimab | CTLA-4 | Chemotherapy+HER2 antagonist | 2024-7-10 | I/II | Open | III/IV | First line | Academic | China |
| 66 | AK112-IIT-C-M-0003 NCT06791148 TrialTroveID-554193 | Low-dose Radiotherapy Combined With Albumin-bound Paclitaxel and AK112 As Second-line Treatment in Patients With Advanced Gastric or Gastroesophageal Junction (G.GEJ) Cancer Who Failed First-line Therapy : A Single, Phase II Trial | Ivonescimab | VEGF | Chemotherapy+Radiation therapy | 2024-7-10 | II | Open | III/IV | Second line | Academic | China |
| 67 | ChiCTR2400087450 TrialTroveID-528449 | A prospective, single-center clinical trial of fruquintinib in combination with cadonilimab and chemotherapy in locally advanced or metastatic adenocarcinoma of the stomach and gastroesophageal junction | Cadonilimab | CTLA-4 | Chemotherapy+Angiogenesis inhibitor | 2024-7-29 | II | Planned | III/IV | First line | Academic | China |
| 68 | LM-302-IIT-203 NCT06587425 TrialTroveID-535875 | A Phase II Study Evaluating the Efficacy and Safety of LM-302 in Combination With Candonilimab and Capecitabine for First-Line Treatment in Patients With Unresectable Advanced, Recurrent, or Metastatic CLDN18.2-Positive Gastric or Gastroesophageal Junction Adenocarcinoma | Candonilimab | CTLA-4 | Chemotherapy+CLDN18.2 antagonist | 2024-7-31 | II | Open | III/IV | First line | Academic | China |
| 69 | ChiCTR2400090935 TrialTroveID-539691 | The Efficacy and Safety of Irinotecan Liposome Injection Combined with Cadonilimab in the Second-line Treatment of Patients with Locally Advanced or Metastatic Gastric Adenocarcinoma | Cadonilimab | CTLA-4 | Chemotherapy | 2024-8-20 | IV | Open | III/IV | Second line | Academic | China |
| 70 | ChiCTR2400089954 TrialTroveID-535831 | A Prospective, Multicenter Clinical Study of the Efficacy and Safety of First-Line Treatment with Ivonescimab in Combination with XELOX for Unresectable Advanced Gastric/Gastroesophageal Junction Adenocarcinoma | Ivonescimab | VEGF | Chemotherapy | 2024-8-31 | II | Planned | III/IV | First line | Academic | China |
| 71 | AK129-103 CTR20242613 NCT06586294 TrialTroveID-535860 | A Phase Ib/II Open Label,Dose Escalation and Dose Extension Study Evaluating the Safety, Tolerability, and Initial Antitumor Efficacy of Anti-PD-1 and Lymphocyte Activation Gene 3(LAG-3) Bispecific Antibody AK129 Combined With Chemotherapy With or Without Cadonilimab in Patients With Human Epidermal Growth Factor Receptor 2 (HER2) Negative Unresectable Locally Advanced or Metastatic G/GEJ Adenocarcinoma | AK129 | LAG3 | Chemotherapy | 2024-9-26 | I/II | Open | III/IV | First line | Industry, all other pharma | China |
| 72 | CIBI363Y110 NCT06610799 TrialTroveID-536645 | Phase Ib Study to Evaluate the Safety, Tolerability and Efficacy of IBI363 in Combination With Oxaliplatin and Capecitabine (XELOX) in First-line Treatment of Unresectable Advanced or Metastatic Gastric and Gastroesophageal Junction Adenocarcinoma | IBI363 | IL2 | Chemotherapy | 2024-10-15 | I | Open | III/IV | First line | Academic | China |
| 73 | ChiCTR2500098646 TrialTroveID-561045 | A Multicenter, Randomized, Controlled, Prospective Clinical Trial on Neoadjuvant Radiotherapy Combined with Cadonilimab, Oxaliplatin, and Capecitabine (XELOX) for Locally Advanced Gastric Cancer | Cadonilimab | CTLA-4 | Chemotherapy+Radiation therapy | 2024-11-19 | II | Planned | III | Neoadjuvant | Academic Industry, all other pharma | China |
| 74 | CTR20244212 CVL006-T1001 NCT06621615 TrialTroveID-537684 | An Open Label, Multicenter Phase I Clinical Study on the Safety, Tolerability, Pharmacokinetics, and Efficacy of CVL006 Injection in Patients With Advanced Solid Tumors. | CVL006 | VEGF | monotherapy | 2024-12-5 | I | Open | III/IV | Second line/Third line | Industry, all other pharma | China |
| 75 | ChiCTR2500096832 TrialTroveID-556206 | Cadonilimab in Combination with Chemotherapy and Chidamide is used in the first-line treatment of Advanced Gastric Cancer | Cadonilimab | CTLA-4 | Chemotherapy | 2024-12-20 | II | Planned | III/IV | First line | Academic | China |
| 76 | ChiCTR2500108850 TrialTroveID-664574 | An exploratory clinical study on the comparison of the efficacy of Kadcyla (AK104) combined with SOX versus Tislelizumab combined with SOX in the first-line treatment of locally advanced or metastatic gastric cancer | Cadonilimab | CTLA-4 | Chemotherapy | 2025-1-1 | II | Open | III/IV | First line | Academic | China |
| 77 | ChiCTR2500103024 TrialTroveID-578614 | An exploratory, single-arm, phase II clinical study of the feasibility of cadonilimab in combination with chemotherapy for the translational treatment of advanced gastric/gastroesophageal conjugate adenocarcinoma | Cadonilimab | CTLA-4 | Chemotherapy | 2025-1-1 | II | Planned | III/IV | First line | Academic | China |
| 78 | 2024-07-077 NCT06630130 Neo-VIKTORY TrialTroveID-538570 | A Phase II Platform Trial of Perioperative Therapies in Locally Advanced Unresectable Gastric Cancer (Neo-VIKTORY) | rilvegostomig | TIGIT | Chemotherapy | 2025-1-22 | II | Open | III | Neoadjuvant | Academic | South Korea |
| 79 | DRAGON-12 NCT06519591 TrialTroveID-527838 | Systemic Application of Cadonilimab, LM-302, and S-1 Combined With Intraperitoneal Infusion of Paclitaxel for the Treatment of Claudin 18.2-positive Gastric Cancer With Peritoneal Metastasis | Cadonilimab | CTLA-4 | Chemotherapy+CLDN18.2 antagonist | 2025-2-25 | II | Open | IV | all lines | Academic | China |
| 80 | NCT06829797 NO.2024-1046 STAR-03 TrialTroveID-557652 | A Multicenter Randomized Controlled Phase II Trial of Iparomlimab and Tuvonralimab (QL1706) Combined with SOX Chemotherapy Versus Chemotherapy Alone in the Treatment of Locally Advanced Gastric or Gastroesophageal Junction Adenocarcinoma(STAR-03) | Iparomlimab/Tuvonralimab | CTLA-4 | Chemotherapy | 2025-2-28 | II | Planned | III/IV | Neoadjuvant | Academic | China |
| 81 | ARTEMIDE-Gastric01 CTR20250520 D702AC00001 EUCT number: 2024-512583-57-00 jRCT2031250011 NCT06764875 TrialTroveID-551822 | A Randomized, Phase Ⅲ Study of Rilvegostomig in Combination With Fluoropyrimidine and Trastuzumab Deruxtecan Versus Trastuzumab, Chemotherapy, and Pembrolizumab for the First Line Treatment of HER2-positive Gastric Cancer (ARTEMIDE-Gastric01) | Rilvegostomig | TIGIT | Chemotherapy+HER2 antagonist | 2025-3-1 | III | Open | III/IV | First line | Industry, Top 20 Pharma | Argentina; Australia; Austria; Belgium; Brazil; Canada; Chile; China; France; Germany; Hong Kong, S.A.R., China; Hungary; India; Italy; Japan; Malaysia; Netherlands; Peru; Poland; Puerto Rico; South Korea; Spain; Taiwan, China; Thailand; Turkey; United Kingdom; United States; Vietnam |
| 82 | NCT06940921 TrialTroveID-571756 Zhang Tao | A Single-arm, Prospective Clinical Study of Low-dose Radiation Therapy (LDRT) Combined With Stereotactic Body Radiation Therapy (SBRT) and Sequential Cadonilimab in the Treatment of Advanced Gastric, Colorectal and Ovarian Cancers With Peritoneal Metastases | Cadonilimab | CTLA-4 | Radiation therapy | 2025-3-20 | I/II | Closed | III/IV | Second line/Third Line/Fourth Line or greater | Academic | China |
| 83 | NCT06766305 QL-GasC-QIBA-1001 TrialTroveID-551855 | Phase Ib/II Clinical Study of QL1706 Combined with SOX Perioperative Treatment for Resectable Locally Advanced Gastric or Gastroesophageal Junction Adenocarcinoma | Iparomlimab/Tuvonralimab | CTLA-4 | Chemotherapy | 2025-3-21 | I/II | Open | II/III | First line | Academic | China |
| 84 | ChiCTR2500106716 NCT06932068 SEARCH TrialTroveID-570195 | Safety and Efficacy of Iparomlimab and Tuvonralimab (QL1706) Combined With Chemotherapy for the Treatment of HER2-Negative, Low PD-L1 Expressing, Unresectable or Metastatic Gastric/Gastroesophageal Junction Adenocarcinoma: A Phase II Single-Arm Trial | Iparomlimab/Tuvonralimab | CTLA-4 | Chemotherapy | 2025-3-26 | II | Open | III/IV | Neoadjuvant | Industry, all other pharma | China |
| 85 | ChiCTR2500108226 TrialTroveID-660400 | A Single-Arm, Phase II Exploratory Study of Short-Course Radiotherapy (SCRT) Sequential to Iparomlimab and Tuvonralimab Combined with SOX Chemotherapy for Neoadjuvant Treatment of Gastric/Gastroesophageal Junction Adenocarcinoma | Iparomlimab and Tuvonralimab | CTLA-4 | Chemotherapy+Radiation therapy | 2025-5-1 | II | Planned | II/III | Neoadjuvant | Academic | China |
| 86 | LanzhouU2H-2025A-017 NCT06949033 TrialTroveID-572880 | Neoadjuvant cadonilimab Combined With Sintilimab and Perioperative SOX Versus Neoadjuvant Sintilimab Combined With Perioperative SOX for Intestinal Type of Locally Advanced Gastric/Gastroesophageal Junction Adenocarcinoma: A Prospective, Multicenter, Open-Label, Randomized Controlled Phase III Study | Cadonilimab | CTLA-4 | Chemotherapy | 2025-5-15 | III | Open | III | Neoadjuvant | Academic | China |
| 87 | ChiCTR2500104237 TrialTroveID-582851 | A single-arm, open, prospective clinical study of Iparomlimab and tuvonralimab in combination with first-line chemotherapy for advanced gastric/gastroesophageal junction adenocarcinoma with low PDL1 expression | Iparomlimab/Tuvonralimab | CTLA-4 | Chemotherapy | 2025-6-1 | II | Planned | III/IV | First line/Second line | Academic | China |
| 88 | NCT07007182 SDZLEC2025-150-02 TrialTroveID-581035 | A Randomized, Controlled, Multicenter Phase II Study of Conversion Therapy Combined With Surgery and Radiotherapy for Retroperitoneal Lymph Node Metastases in Gastric Cancer | IBI318 | PD-L1 | monotherapy | 2025-7-1 | II | Planned | III | First line | Academic | Hong Kong |
| 89 | NCT07053332 neoCACR for dMMR/MSI-H G/GEJ TrialTroveID-644444 | A Single-arm, Prospective Phase II Clinical Study of Neoadjuvant PD-1/CTLA-4 Combination Antibody With Low-dose Radiotherapy in Resectable dMMR/MSI-H Esophagogastric Junction/Gastric Adenocarcinoma. | Iparomlimab/Tuvonralimab | CTLA-4 | Chemotherapy | 2025-7-1 | II | Planned | II/III | Neoadjuvant | Academic | Thailand |
| 90 | ChiCTR2500106665 JS-GI 2501 TrialTroveID-650507 | Assessment of the efficacy and safety of Apatolimab Tovolimab in combination with regorafenib and paclitaxel as second-line treatment for patients with advanced gastric or gastroesophageal junction (G/GEJ) adenocarcinoma who have failed immunotherapy combined with chemotherapy: An open-label, multicenter Phase II clinical study | Iparomlimab/Tuvonralimab | CTLA-4 | Chemotherapy+Angiogenesis inhibitor | 2025-7-15 | II | Planned | I/II/III/IV | Second line | Academic | China |
| 91 | CTR20252412 D9804C00001 EUCT number: 2024-516909-23-00 GEMINI-PeriOp Gastric GEMINI-PeriOp GC NCT07069712 TrialTroveID-647413 | A Master Protocol of an Open-Label, Multi-Drug, Multi-Center, Phase II Platform Study to Evaluate the Safety, Tolerability, Pharmacokinetics, and Preliminary Anti-tumor Activity of Novel Agents or Combinations as Perioperative Treatment in Participants With Locally Advanced Resectable Gastroesophageal Adenocarcinoma (GEMINI-PeriOp GC) | rilvegostomig | TIGIT | Chemotherapy | 2025-7-17 | II | Open | III | Neoadjuvant | Industry, Top 20 Pharma | Canada; China; Georgia; Italy; Japan; Poland; Spain; Taiwan, China; Turkey; United Kingdom; United States |
| 92 | K7937 NCT07091227 TrialTroveID-650658 | Efficacy and Safety of AK112 Combined Chemotherapy as Neoadjuvant Treatment for Signet Ring Cell-containing Gastric or Gastroesophageal Junction Adenocarcinoma | Ivonescimab | VEGF | Chemotherapy | 2025-7-20 | II | Planned | III | Neoadjuvant | Academic | China |
| 93 | ChiCTR2500106399 TrialTroveID-649112 | Neoadjuvant Cadonilimab Combined With Perioperative SOX Versus Neoadjuvant Sintilimab Combined With Perioperative SOX for Diffuse Type of Locally Advanced Gastric/Gastroesophageal Junction Adenocarcinoma:A Prospective, Multicenter, Open-Label, Randomized Controlled Phase II Study | Cadonilimab | CTLA-4 | Chemotherapy | 2025-7-23 | II | Planned | III | Neoadjuvant | Academic | China |
| 94 | 2025 (64-01) NCT07165847 TrialTroveID-664748 TRIUNITE-05 | Neoadjuvant Radiotherapy Plus Tegafur, Oxaliplatin and Iparomlimab and Tuvonralimab in Resectable Gastric and GE-junction Cancer : A Randomized, Two-arm, Prospective Trial (TRIUNITE-05) | Iparomlimab/Tuvonralimab | CTLA-4 | Chemotherapy+Radiation therapy | 2025-7-23 | II | Open | II/III | Neoadjuvant | Academic | China |
| 95 | NCT07045805 QL-GasC-QIBA-1002 TrialTroveID-642311 | Phase II Study of Iparomlimab and Tuvonralimab Combined With Paclitaxel and Bevacizumab for the Treatment of Advanced Gastric or Gastroesophageal Junction Adenocarcinoma That Failed First-Line Treatment With PD-(L)1 Inhibitor Combined With Chemotherapy | Iparomlimab/Tuvonralimab | CTLA-4 | Chemotherapy+angiogenesis inhibitor | 2025-7-25 | II | Planned | IV | Second line | Academic | China |
| 96 | CTR20252338 NCT07022002 SSGJ-705-201 TrialTroveID-638902 | A Phase II Study to Evaluate the Safety and Efficacy of SSGJ-705 Monotherapy and Combination Therapy in Patients With Advanced HER2-Expressing Solid Tumors | SSGJ-705 | HER2 | Chemotherapy | 2025-7-30 | II | Planned | III/IV | First line/Second line | Industry, all other pharma | China |
| 97 | AK104-310 AK104-310/COMPASSION-33 CTR20252052 NCT07023315 TrialTroveID-638940 | A Randomized, Double-blind, Phase III Clinical Study Comparing the Efficacy and Safety of Cadonilimab Plus Oxaliplatin and Tegafur-Gimeracil-Oteracil Potassium (SOX) Versus Placebo Plus SOX as Perioperative Treatment for Patients With Resectable Gastric and Gastroesophageal Junction (G/GEJ) Adenocarcinoma | Cadonilimab | CTLA-4 | Chemotherapy | 2025-8-7 | III | Open | I/II | Neoadjuvant | Industry, all other pharma | China |
| 98 | AK112-IIT-003 NCT06904300 TrialTroveID-565494 | A Randomized, Controlled, Multi-center Phase II Study of Ivonescimab (AK112) Plus Paclitaxel Versus Paclitaxel With or Without Ramucirumab as Second-line Therapy in Subjects With Advanced Gastric or Gastroesophageal Junction（G.GEJ）Cancer Who Failed Immunochemotherapy | Ivonescimab | VEGF | Chemotherapy | 2025-8-15 | II | Planned | III/IV | Second line | Academic | China |
| 99 | CTR20253351 NCT07142837 TrialTroveID-659844 ZG005-JAK-004 | A Study to Evaluate the Efficacy and Safety of ZG005 in Combination With Gecacitinib and Chemotherapy for Participants With Advanced Solid Tumors | ZG005(nilvanstomig) | TIGIT | Chemotherapy | 2025-9-1 | I/II | Planned | III/IV | Second line | Industry, all other pharma | China |
| 100 | 2023-01 EUCT Number: 2023-503451-94-00 EudraCT Number: 2023-503451-94 IB2023-01 NCT05888857 TAYLOR TrialTroveID-472492 | A Multicentric Phase II Trial Evaluating MEDI5752 in Patients With Mature Tertiary Lymphoid Structures Solid Tumors. | MEDI5752 | CTLA-4 | Chemotherapy | 2025-9-1 | II | Planned | III/IV | First line/Second line | Academic | France |
| 101 | 2025-399 NCT07139587 TrialTroveID-659162 | The Efficacy and Safety of Iparomlimab/Tuvonralimab (Anti PD-1/CTLA-4) Combined With Albumin-bound Paclitaxel in Second-line Treatment of Patients With Advanced Gastric/Gastroesophageal Junction Adenocarcinoma | Iparomlimab/Tuvonralimab | CTLA-4 | Chemotherapy | 2025-9-1 | II | Planned | III/IV | Second line | Academic | China |
| 102 | NCT07151209 QL-GasC-QIBA-3011 TrialTroveID-662183 | A Prospective, Multicenter, Open-label Phase II Single-arm Clinical Trial Protocol on the Efficacy and Safety of Lparomlimab and Tuvonralimab in Combination With Lenvatinib and SOX Chemotherapy in Potentially Resectable MSI-H, dMMR Locally Advanced Gastric or Gastroesophageal Junction Adenocarcinoma Patients | Iparomlimab/Tuvonralimab | CTLA-4 | Chemotherapy+Angiogenesis inhibitor | 2025-9-1 | II | Planned | III/IV | First line | Academic | China |
| 103 | NCT07127822 PUCH MSI-H GC 1st TrialTroveID-656739 | A Randomized, Controlled, Non Inferiority Phase II Clinical Study Comparing Iparomlimab and Tuvonralimab With Standard Chemotherapy Combined With PD-1/PD-L1 Monoclonal Antibody as First-line Treatment for MSI-H/dMMR Recurrent/Metastatic Gastric Cancer | Iparomlimab/Tuvonralimab | CTLA-4 | Chemotherapy | 2025-9-1 | II | Planned | III/IV | Second line | Academic | China |
| 104 | CTR20253553 CVL006-T1002 NCT07157956 TrialTroveID-663023 | Phase I/II Clinical Study of CVL006 Combination Therapy in Advanced Solid Tumors | CVL006 | VEGF | Chemotherapy | 2025-9-10 | I/II | Open | III/IV | Second line | Industry, all other pharma | China |
| 105 | NCT07148427 QL-GasC-QIBA-3007 TrialTroveID-660536 | A Single-arm, Phase II Clinical Study Protocol of Iparomlimab and Tuvonralimab in Combination With Regorafenib and Chemotherapy as First-line Treatment for Locally Advanced or Metastatic Gastric/Gastroesophageal Junction Adenocarcinoma | Iparomlimab/Tuvonralimab | CTLA-4 | Chemotherapy+Angiogenesis inhibitor | 2025-9-20 | II | Planned | III/IV | First line | Academic | China |
| 106 | 2025KY011 NCT07289997 TrialTroveID-686901 | Iparomlimab and Tuvonralimab Injection Combined With Apatinib and Irinotecan Hydrochloride for the Treatment of Advanced Alpha-fetoprotein-producing Gastric Cancer (AFPGC) That Progresses From First-line PD-1 Combined Chemotherapy, A Prospective, Single-arm, Phase II Clinical Study | Iparomlimab and Tuvonralimab | CTLA-4 | Chemotherapy+Angiogenesis inhibitor | 2025-9-28 | II | Open | III/IV | Second line | Academic | China |
| 107 | 25-327 NCT07070466 TrialTroveID-647436 | A Single-Arm, Phase II Study of Ivonescimab in Combination With FOLFOX in Advanced HER2 Negative Gastroesophageal Adenocarcinomas | Ivonescimab | VEGF | Chemotherapy | 2025-10-10 | II | Open | III/IV | First line | Academic | United States |
| 108 | CTR20253728 NCT07233850 SSGJ-706-202 TrialTroveID-684129 | A Phase II Clinical Study Evaluating SSGJ-706 in Combination Therapy for Advanced Gastrointestinal Cancers | SSGJ-706 | PD-L1 | Chemotherapy | 2025-10-27 | II | Open | III/IV | First line | Industry, all other pharma | China |
| 109 | BFHHS20250012 NCT07315854 TrialTroveID-688221 | A Prospective, Single-Center, Exploratory Phase II Clinical Study of Iparomlimab and Tuvonralimab (QL1706) Combined With Chemotherapy in the Treatment of Previously Untreated Advanced or Metastatic Gastric or Gastroesophageal Junction Cancer | Iparomlimab/Tuvonralimab | CTLA-4 | Chemotherapy | 2025-11-4 | II | Open | III/IV | First line | Academic | China |
| 110 | EUCT Number: 2025-520694-39-00 GRACIE NCT06846346 TrialTroveID-559122 UC-GIG-2411 | Phase II Study Evaluating Ivonescimab in Combination With Chemotherapy for First- and Second-line Treatment of Advanced or Metastatic Gastric and Gastroesophageal Adenocarcinoma Patients | Ivonescimab | VEGF | Chemotherapy | 2025-11-20 | II | Open | III/IV | First line/Second line | Cooperative Group | France |
| 111 | NCT07315035 QILIN-01 TrialTroveID-688326 | A Randomized, Controlled, Phase-II Trial of Neoadjuvant QL1706 (Iparomlimab /Tuvonralimab) Combined With SOX Versus SOX Alone Followed by Curative Gastrectomy in Patients With Locally Advanced Diffuse-Type Gastric Adenocarcinoma | Iparomlimab/Tuvonralimab | CTLA-4 | Chemotherapy | 2026-1-1 | II | Planned | III | Neoadjuvant | Academic | China |
| 112 | CLARITY-Gastric 02 CTR20254901 CTR20254902 D9803C00001 EUCT number: 2024-519787-40-00 NCT07431281 TrialTroveID-688507 | A multicenter, randomized, controlled phase III study (CLARITY-Gastric 02) evaluating Sonesitatug vedotin in combination with fluoropyrimidine and or without Rilvegostomig as first-line treatment in subjects with Claudin18.2 positive, HER2 negative, advanced/metastatic gastric, gastroesophageal junction, or esophageal adenocarcinoma. | Rilvegostomig | TIGIT | Chemotherapy+CLDN18.2 antagonist | 2026-2-3 | III | Open | III/IV | First line | Industry, Top 20 Pharma | Australia; Austria; Belgium; Brazil; Canada; China; France; Germany; Hungary; India; Italy; Japan; Netherlands; Poland; Puerto Rico; South Korea; Spain; Thailand; Turkey; United Kingdom; United States |
| 113 | 2025-523590-42-00 ASCEND CR-001-101 EUCT number: 2025-523590-42-00 NCT07335497 TrialTroveID-689214 | A Phase I/II, Multicenter, Open-label, Dose Escalation and Dose Expansion Study to Evaluate the Safety, Pharmacokinetics, Pharmacodynamics, and Antitumor Activity of CR-001 in Adult Participants With Locally Advanced or Metastatic Solid Tumors | CR-001 | VEGF | monotherapy | 2026-2-17 | I/II | Open | III/IV | First line/Second line | Industry, all other pharma | United States |
| 114 | CIBI363GC002 NCT07361991 TrialTroveID-690253 | A Phase Ib/II Study to Evaluate the Safety and Efficacy of IBI363 in Combination With Bevacizumab With or Without Nab-Paclitaxel as Second-Line Therapy in Patients With Advanced Gastric Cancer | IBI363 | IL2 | Chemotherapy+Angiogenesis inhibitor | 2026-3-1 | I/II | Planned | III/IV | Second line | Academic | China |
| 115 | NCT07400315 QL-GasC-QIBA-3035 TrialTroveID-692089 | A Prospective, Single-arm, Multicenter, Phase II Clinical Study of Iparomlimab and Tuvonralimab (QL1706) in Combination With Modified FLOT Regimen (TFOX) as First-line Treatment for HER2-negative Advanced Gastric or Gastroesophageal Junction Adenocarcinoma | Iparomlimab and Tuvonralimab | CTLA-4 | Chemotherapy | 2026-3-1 | II | Planned | III/IV | First line | Industry, all other pharma | Hong Kong |
| 116 | CA266-0004 CA2660004 CTR20260794 EudraCT Number: 2025-523263-37 NCT07221149 ROSETTA Gastric-204 ROSETTA GI 204 TrialTroveID-680398 U1111-1325-8116 | ROSETTA Gastric-204: A Blinded, Randomized, Phase II/III Study of Pumitamig in Combination With Chemotherapy Versus Nivolumab in Combination With Chemotherapy in Participants With Previously Untreated Advanced or Metastatic Gastric, Gastroesophageal Junction, or Esophageal Adenocarcinoma | Pumitamig | VEGF | Chemotherapy | 2026-3-13 | II/III | Open | III/IV | First line | Industry, Top 20 Pharma | Argentina; Australia; Brazil; Canada; Chile; China; Colombia; France; Germany; India; Italy; Japan; Mexico; Poland; Romania; South Korea; Spain; Turkey; United Kingdom; United States |
| 117 | AK104-SC-01 CTR20260795 NCT07449780 TrialTroveID-695542 | A Randomized, Open-label, Multicenter Phase I/III Clinical Trial Evaluating the Pharmacokinetics, Efficacy, and Safety of AK104 (SC) in Combination with Oxaliplatin and Capecitabine(XELOX) Versus AK104 (IV) in Combination with XELOX as First-line Treatment for Unresectable Locally Advanced or Metastatic Gastric Adenocarcinoma or Gastroesophageal Junction Adenocarcinoma. | Cadonilimab | CTLA-4 | Chemotherapy | 2026-3-18 | III | Open | III/IV | First line | Industry, all other pharma | China |
| 118 | CTX-10726-001 NCT07419841 TrialTroveID-693085 | A Phase I, Open-Label, Multiple-Ascending Dose Study of the Safety and Tolerability of CTX-10726 in Patients With Advanced Malignancies | CTX-10726 | VEGF | monotherapy | 2026-4-1 | I | Open | III/IV | First line/Second line | Industry, all other pharma | United States |
| 119 | NCT07453394 QLS5132-201 TrialTroveID-694797 | A Phase Ib/II Clinical Study on the Safety, Tolerability, Pharmacokinetics, and Efficacy of Intravenous QLS5132 Combination Therapy in Participants With Advanced Solid Tumors | Iparomlimab/Tuvonralimab | CTLA-4 | CLDN18.2 antagonist | 2026-4-1 | I/II | Planned | III/IV | all lines | Industry, all other pharma | Thailand |
| 120 | CTR20260332 LBL-024-CN004_04 NCT07390383 TrialTroveID-691566 | An Open-label, Multicenter, Phase II Clinical Study to Evaluate the Efficacy and Safety of LBL-024 in Combination With Other Drugs for the Treatment of Patients With Advanced Solid Tumour[Substudy Number 04] | LBL-024 | 4-1BB | Chemotherapy | 2026-4-1 | II | Planned | III/IV | all lines | Industry, all other pharma | China |
| 121 | NCT07502027 TJ-IRB202602008 TrialTroveID-697815 | A Multicenter, Single-arm, Exploratory Clinical Study of Iparomlimab and Tuvonralimab Combined With SOX Following Heterogeneous Radiotherapy as First-line Treatment for Unresectable Locally Advanced or Metastatic HER2-negative Gastric or Gastroesophageal Junction Adenocarcinoma | Iparomlimab and Tuvonralimab | CTLA-4 | Chemotherapy+Radiation therapy | 2026-6-1 | IV | Planned | III/IV | First line | Academic | China |

**Supplementary Table S2** The results of time trend analysis

| Variable | Years used | Coefficient | P value | Trend direction |
| --- | --- | --- | --- | --- |
| Total trial counts | 2017-2025 | 0.3837 | 0.0000 | Increasing |
| Phase I | 2017-2025 | -0.0316 | 0.7225 | Decreasing |
| Phase I/II | 2017-2025 | 0.2240 | 0.0189 | Increasing |
| Phase II | 2017-2025 | 0.7853 | 0.0000 | Increasing |
| Phase II/III | 2017-2025 | 0.1549 | 0.5091 | Increasing |
| Phase III | 2017-2025 | 0.6837 | 0.0358 | Increasing |
| Phase IV | 2017-2025 | 0.3461 | 0.2123 | Increasing |
| CTLA-4 | 2017-2025 | 0.4560 | 0.0000 | Increasing |
| VEGF | 2017-2025 | 0.5973 | 0.0024 | Increasing |
| TIGIT | 2017-2025 | 0.4806 | 0.0159 | Increasing |
| Others | 2017-2025 | 0.1240 | 0.1550 | Increasing |

**Supplementary Table S3** Comparison of characteristics between clinical trials with and without China participation

| Characteristics | Cases | Country | | *χ^2^* | *P* |
| --- | --- | --- | --- | --- | --- |
|  |  | China, *n* | Non-China, *n* |  |  |
| Trial phase |  |  |  | 1.245 | 0.462 |
| Phase I, I/II, II | 105 | 87 | 18 |  |  |
| Others | 16 | 15 | 1 |  |  |
| Trial status |  |  |  | 4.021 | 0.060 |
| Completed | 15 | 10 | 5 |  |  |
| Others | 106 | 92 | 14 |  |  |
| Co-target |  |  |  | 2.044 | 0.153 |
| CTLA-4 | 75 | 66 | 9 |  |  |
| Others | 46 | 36 | 10 |  |  |
| Co-target |  |  |  | 1.981 | 0.232 |
| VEGF | 14 | 10 | 4 |  |  |
| Others | 107 | 92 | 15 |  |  |
| Therapeutic regimen |  |  |  | 8.672 | 0.007 |
| Monotherapy | 22 | 14 | 8 |  |  |
| Combination therapy | 99 | 88 | 11 |  |  |
| Patient stage |  |  |  | 0.011 | 1.000 |
| I/II, II/III | 7 | 6 | 1 |  |  |
| Others | 114 | 96 | 18 |  |  |
| Treatment line |  |  |  | 1.661 | 0.198 |
| Neoadjuvant/First line | 70 | 58 | 12 |  |  |
| Others | 51 | 44 | 7 |  |  |
| Sponsor type | |  |  | 2.582 | 0.108 |
| Academic | 65 | 58 | 7 |  |  |
| Others | 56 | 44 | 12 |  |  |
|  | | | | | |

**
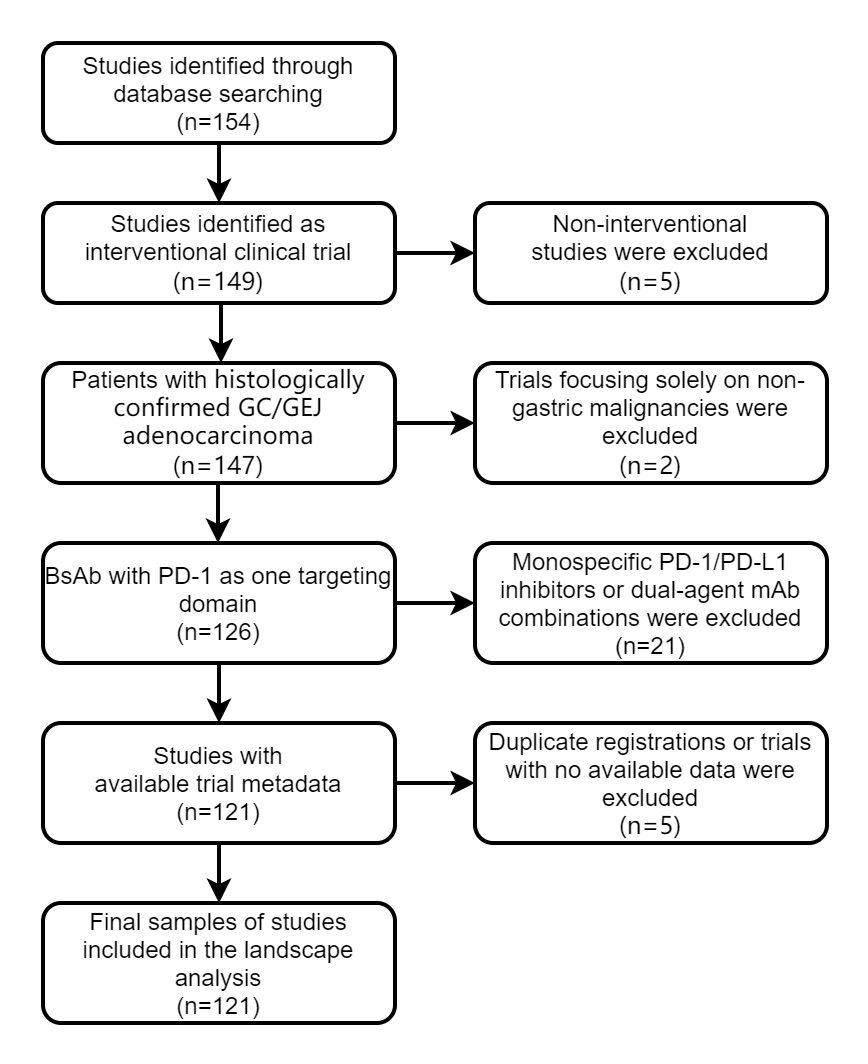
Supplementary Figure S1** Flowchart of clinical trial selection for PD-1-containing bispecific antibodies in gastric cancer.

**
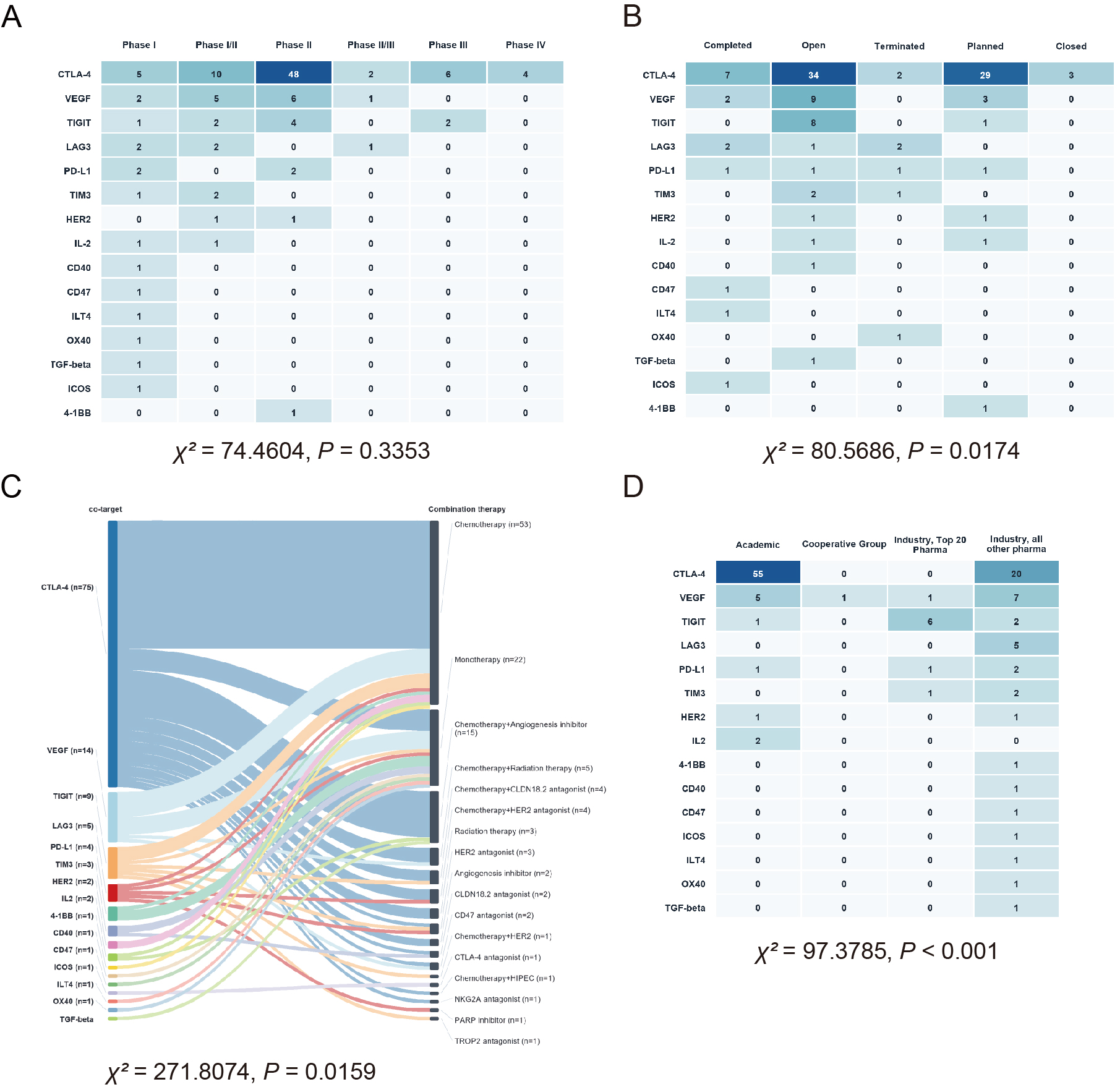
****Supplementary Figure S2** Co-target correlation analysis. **(A).** Association between co-targets and trial phase; **(B).** Association between co-targets and trial status; **(C).** The Sankey diagram showing co-targets to combination therapy; **(D).** Association between co-target and sponsor type.
